# Structural and Magneto-Optical Characterization of La, Nd: Y_2_O_3_ Powders Obtained via a Modified EDTA Sol–Gel Process and HIP-Treated Ceramics

**DOI:** 10.3390/ma13214928

**Published:** 2020-11-02

**Authors:** Andrzej Kruk

**Affiliations:** Institute of Technology, Pedagogical University, Podchorążych 2, 30-084 Kraków, Poland; andrzej.kruk@up.krakow.pl

**Keywords:** transparent ceramics, Y_2_O_3_, magneto-optical properties, EDTA sol–gel process, Verdet constant

## Abstract

In this study, pure Y_2_O_3_, La_0.1_Y_1.9_O_3_ and La_0.1_Nd_0.12_Y_1.78_O_3_ nanosized powders were successfully synthesized by a modified sol–gel method. Pure and rare-earth ions doped yttria powders were characterized by X-ray diffraction, scanning electron microscopy and Brunauer–Emmett–Teller methods. The powders were sintered by the hot isostatic pressing process. The highest in-line transmittance of 56% was obtained at 800 nm and increased in the IR region. The influence of the lanthanum and neodymium ions on the physicochemical properties of yttria were discussed. The La-Nd-doped material exhibited a Verdet constant over 4000 deg/T·m at 400 nm and low thermal dependence. An interesting evolution of the Verdet constant across the absorption band with high resolution was studied. A study of the optical and magneto-optical properties of yttria doped with Nd^3+^ and La^3+^ is discussed in this paper.

## 1. Introduction

In recent years, extensive efforts have been made to develop transparent materials based on the cubic oxides RE_2_O_3_ (RE = Y, Sc, Lu or Gd) due to the strong demand for applications, such as solid-state laser materials [1], sight windows [2], scintillators [3], nuclear medicine [4], X-ray computed tomography [5], security [6], astrophysics [7] and particle physics [8]. Yttria (Y_2_O_3_) is commonly used as a host lattice to incorporate optical activator ions because of its high thermal (2700 K melting point) and chemical stability and wide optical band gap (5.5 eV). The cubic-Y_2_O_3_ crystalline phase indicates a large UV–IR transmittance (0.280–8 mm) and low coefficient of thermal expansion. Doped rare-earth Y_2_O_3_ elements have been used as a host lattice for manufacturing lasers, optical fiber scintillators, windows and optical isolators in communications as well as phosphors [9]. Due to its high dielectric constant, yttria may be used in devices for the semiconductor components of metal oxide semiconductors (MOSs) [10] and flat-panel display applications such as light-emitting diode (LED), field-emission display (FED) and photodiode (PD) transistors [11]. This paper reports the fabrication and operation of pure and rare-earth ions doped Y_2_O_3_ as a potential magneto-optical material to use as a host material in magneto-optical devices. Pure and metal-doped Y_2_O_3_ nanopowders have been produced via several methods. Some recent techniques include thermal decomposition [11], the hydrothermal [12], combustion [13] and citrate gel methods [14], spray pyrolysis [15] the urea method [16], coprecipitation [16] and mechanochemical processing [17]. Wet chemical methods like coprecipitation and sol–gel provide a high degree of homogeneity, uniform particle size and purity [7]. Dense and transparent materials can be obtained by using different techniques, such as hot pressing [18], hot isostatic pressing (HIP) [19], vacuum sintering [20], self-propagating high-temperature synthesis (SHS) [21] and arc plasma synthesis [22]. 

Rare-earth elements have unpaired 4f electrons and unfilled 4f shells, indicating high paramagnetic properties. Additionally, their outer 4f electrons can be easily excited to the higher energy of the 5d orbit due to the transition between 4f^8^ and 4f^7^5d under the magnetic field, which leads to a strong Faraday effect [23,24]. Complex theoretical approaches have been performed through the Hamiltonian perturbation, which takes into consideration the crystal field, spin–orbit coupling and superexchange interaction [25]. Magneto-optical devices or magnetic field sensors are commonly used in many applications, such as optical communications, high-power laser systems, diluted magnetic semiconductors, optical modulators and optical parameter amplifiers [26,27,28,29]. They are based on the Faraday phenomena, which manifest the change of angle of polarized light passing through a transparent sample placed parallel to an external magnetic field [25]. Recently, pure and doped crystals, such as yttrium aluminate garnet (Y_3_Al_5_O_12_ YAG), yttrium iron garnet (Y_3_Fe_5_O_12_ YIG) or terbium scandium aluminum garnet (Tb_2_ScAl_4_O_12_ TSAG), have been commonly used as magneto-optical materials. Rare-earth elements like Dy [29], Pr, Ce [30] and Tb [31,32] in crystals and yttrium oxide ceramics increase the Verdet constant. The above-mentioned crystals exhibit excellent physical properties, such as a high Verdet constant and high transparency (up to 90%) in the 1500–5000 nm IR region [33,34,35]. However, pure and doped with rare-earth element crystals are not useful because of their absorption bands in the visible and near-infrared spectral region [35]. Furthermore, the production of crystals is also very expensive and the average crystal size is not enough to use in magneto-optical devices, which work with high-power lasers [32,34]. A principal advantage of sinters is their optical and magneto-optical isotropy. Results indicate that the doping of La^3+^ ions improves the intensity of emission bands in the near-infrared (NIR) region and decreases the calcination temperature [35]. Both La and Nd ions were selected because they display many interesting physicochemical properties. Powders were prepared by a modified EDTA (C_10_H_16_N_2_O_8_) sol–gel method. Bulk samples were prepared by the HIP method. The effect of La and Nd ions on the structural and the magneto-optical and properties of nanocrystalline Y_2_O_3_ sinters were investigated by X-ray diffraction, scanning electron microscopy (SEM) with energy-dispersive spectroscopy (EDS), Fourier transform infrared spectroscopy (FT-IR), Raman spectroscopy and temperature-dependent magneto-optical spectroscopy. 

These results contain valuable data concerning the magneto-optical properties of La and La, Nd-Y_2_O_3_ translucent ceramics presenting an evolution of the Verdet constant across the absorption band with a high resolution. A detailed correlation between structures, microstructural and magneto-optical characterization and their property evaluation will be presented and discussed. 

## 2. Experimental 

The particle size and morphology of the ceramics were investigated with the JEOL6610LV SEM (Tokyo, Akishima, Japan) coupled with an Oxford EDS analyzer. In order to determine the grain size distribution of the sintered pellets, a series of SEM images were processed using the ImageJ version 1.14 software following binarization.

The phase structure and composition of ceramics were characterized via X-ray diffraction (XRD X′Pert Panalytical) (Malvern, Worcestershire, UK) with Cu(K*_α_*) radiation. The XRD data were analyzed using the (Highscore Plus Panalytical, version 3.0) software with Rietveld’s algorithm to specify the structural parameters. The standard dataset of PCPDFWIN version 2.3 was used for data analysis.

The specific surface area of the powders was measured by using the low-nitrogen BET adsorption method (ASAP 2010 version 4.00 G). 

The pycnometer density of the powder, which provides the theoretical density of the powder, was determined according to the ASTM D 4892 standard. 

The lattice spacing (d_hkl_) was computed using Bragg’s law, and the crystallite sizes (*D_XRD_*) of powders and bulk materials were calculated using the standard Scherrer equation [36]:

(1) $$D_{XRD}=\frac{K\times \lambda }{\beta \times cos\theta }$$

where *K* = 0.89, *λ* is the wavelength of Cu-K*α* radiation, *β* is the corrected full width at half-maximum (FWHM) of the diffraction peak (in radians) and *θ* is the diffraction angle. *β* is the width of the specimen’s peak corrected by an instrumental broad factor:

(2) $$\beta ={\left(B_{obs}^{2}-B_{abs}^{2}{}^{}\right)}^{1/2}$$

where *B_obs_* is the FWHM, which is related to the sample, and *B_abs_* is the FWHM of the standard (Al_2_O_3_). 

The particle sizes *D*_BET_ (nm) of the powders were calculated to the following formula:

(3) $$D_{BET}=\frac{6\times 10^{3}}{S_{BET}\times \rho }$$

where *S*_BET_ is a parameter of the BET specific surface area (cm^2^/g) and *ρ* is the theoretical density of the nanosized powders (Y_2_O_3_ 5.031: g/cm^3^, La_0.1_Y_1.9_O_3_: 5.026 g/cm^3^, La_0.1_Nd_0.12_Y_1.78_O_3_: 5.018 g/cm^3^).

The average dislocation density of the particulate structure can be computed using the relation:

(4) $$\rho \approx \frac{1}{{\left(Dxrd\right)}^{2}}$$

The Horiba LabRam HR800 Spectrometric Analyzer was used to obtain Raman spectra in the range of 300–4850 cm^−1^ with a 1.5 cm^−1^ step and a laser light source at 532 nm. Raman spectra were performed on bulk samples.

Luminescence was excited by the CW 808 nm diode laser (Spectra Laser, Opole, Opolskie, Poland). The emission signal from the sample was recorded using the SilverNova Stellarnet (Tampa, FL, USA) spectrometer.

The Fluorolog Tau-3 Lifetime System by Horiba (Kyoto, Japan) was used to measure lifetimes. For the excitation sample, a solid-state 480 nm laser was used.

The above-mentioned experiments were conducted at room temperature.

Absorbance coefficient spectra were obtained with the SilverNova Stellarnet spectrometer in the 300–1100 range with a 0.5 nm step at selected temperatures: 294, 304, 314, 324 and 334 K.

To measure the Verdet constant, a dedicated spectrometer was used. Details were presented in a previous paper by the author of [37]. 

The temperature dependence of the absorbance coefficient and Faraday rotation and its causes were also investigated in the range of 294–334 K with a 10 K step. 

The composition, shape and dimensions of each sample are summarized together in Table 1.

### Preparation of Powders

The pure Y_2_O_3_ nanosized powder and Y_2_O_3_ doped with 10 mol.% La^3+^ ions or 10 mol.% La^3+^ and 12 mol.% Nd^3+^ were prepared by a modified EDTA sol–gel technique. The high-purity metal oxide, yttrium oxide (>99.9%, Sigma Aldrich), lanthanum nitrate (>99.9%, Sigma Aldrich), neodymium nitrate (>99.9%, Sigma Aldrich)(Kenilworth, NY, USA), nitric acid (POCH, Gliwice, Górny Śląsk, Poland), ammonia (65%, POCH) and ethylenediaminetetraacetic acid (EDTA, 99.995%, Aldrich) were used as starting materials. EDTA is an aminopolycarboxylic acid and water-soluble solid. When used as a chelating agent, it reduces the concentration of free-metal ions in the precursor. The conditional stability constant β_m_, defined as the relation between the free ions in the solution and the concentration of complexing ions of the metal in the equilibrium state, provides a measure of the stability of these complexes. As a chelating agent, it is able to chelate multivalent cations, representing stability in the binding strength between the chelate and metal ions (cations) [38,39]. These metal nitrate solutions and yttrium powders were precisely weighed in the required proportions according to the assumed chemical compound. The yttrium oxide powder was dissolved in aqueous nitric acid to prepare the nitrate solution. In the next step, metal nitride oxides were mixed and added to the 0.1 M EDTA acid water solution. The molar ratio of metal ions to hydroxyl was 1:1.5. The synthesis of the liquid precursor resulted in the formation of stable complexing ions with the metal cations of yttrium, lanthanum and neodymium. The solution was continuously mixed and an ammonia solution was slowly dropped to obtain a solution with pH 7.5. The mixture was stirred and heated at 773 K in ambient air to form a transparent gel by the removal of excess moisture. The calcination temperature in the modification process was decreased in order to reduce the impurity and improve the crystallinity of the powders. The gels were calcined at 953 K in air. After the calcination process, the obtained powder was milled (2 h in ethanol with zirconia balls) in a rotary mill. The powders were formed into green bodies via biaxial pressing under a pressure of 80 MPa. The green bodies received additional cold isostatic pressing under 250 MPa. The samples were deparaffinated and sintered by hot isostatic pressing for 2 h in argon at 1730 K and under a uniaxial pressure of 30 MPa. Finally, the samples were polished to a mirror-like finish on both sides in order to reduce the impact artifacts such as light scattering and/or diffraction.

The EDTA gel processes combined with the appropriate thermal treatment can be used to obtain fully reacted nanophosphors with the desired phase and chemical composition. This method is noneffective for the synthesis of a large number of products.

## 3. Results and Discussion

### 3.1. X-Ray Diffraction of Powders 

**Figure 1:**
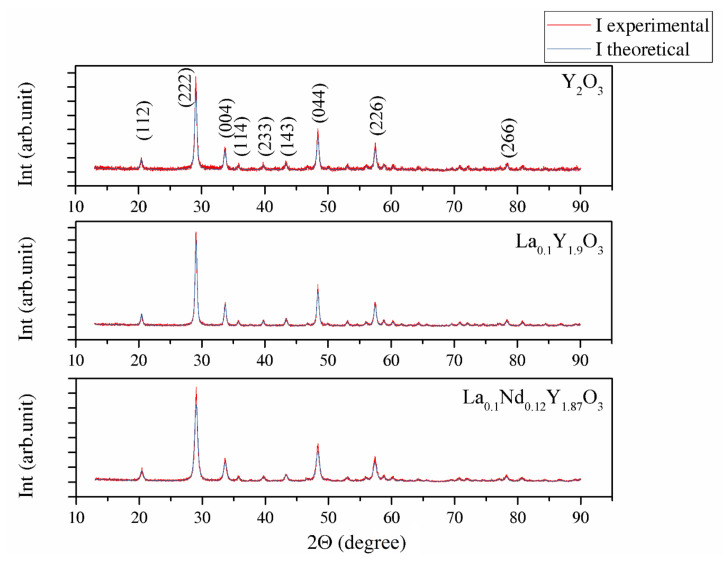
XRD patterns of Y_2_O_3_, La_0.1_Y_1.9_O_3_ and La_0.1_Nd_0.12_Y_1.78_O_3_ phosphors obtained via EDTA gel processes after calcination at 973 K.

Figure 1 shows the XRD patterns of Y_2_O_3_, La_0.1_Y_1.9_O_3_ and La_0.1_Nd_0.12_Y_1.78_O_3_ phosphors obtained via EDTA gel processes following calcination at 953 K. The powders show a crystalline pattern in which the diffraction peaks correspond to the full crystalline cubic phase of Y_2_O_3_ (JCPDS Card No. 98-008-1861) with the Ia-3 space group. No secondary phase was found, proving the complete incorporation of rare elements (RE) into the yttria crystal lattice. The theoretical model (*I*_the_) was fitted to the experimental XRD data (*I*_exp_). The average crystallite size *D_XRD_*, lattice constant *a*, unit cell volume *V*, average dislocation density *ρ* and *BET* of powders parameter are presented together in Table 2. The particle sizes of *D*_BET_ for the Y_2_O_3_, La_0.1_Y_1.9_O_3_ and La_0.1_Nd_0.12_Y_1.78_O_3_ nanopowders are 371.6, 368.1 and 467.1 nm, respectively. The particle size obtained from the BET specific surface area is approximately 20 times larger than the crystallite size obtained from the XRD method, indicating the existence of low agglomeration among particles. The volume of the unit cell for the doped powders increased because of the presence of La and Nd, which exhibited larger ionic radii, consistent with the theoretical predictions. Figure 1 shows that the Bragg peak positions shift to lower *2Θ* positions with RE concentrations. This result indicates the change (stress) of the host lattice due to the substitution of Y^3+^ (0.089 nm) ions with larger La^3+^ (0.103 nm) and Nd^3+^ (0.108 nm) ions. It was also shown that La^3+^ ions influence the increase in the RE’s luminescence level because the local symmetry of the crystal field around RE^3+^ reduces [40].

### 3.2. SEM Observation of Powders 

**Figure 2:**
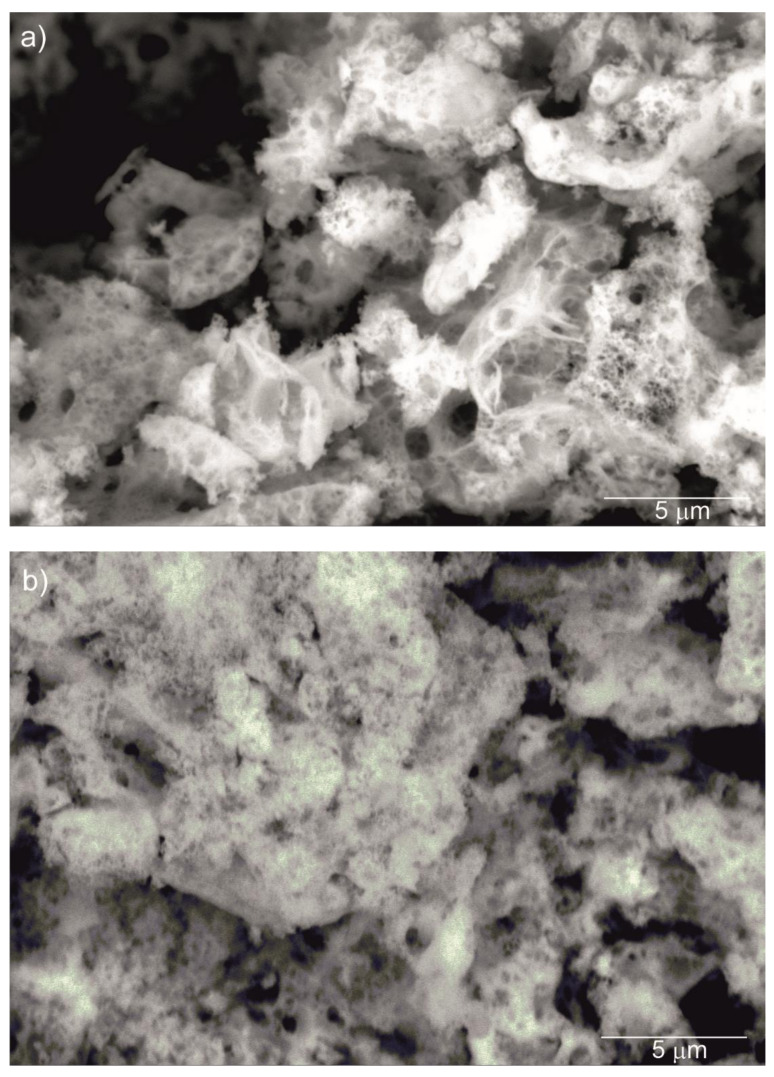

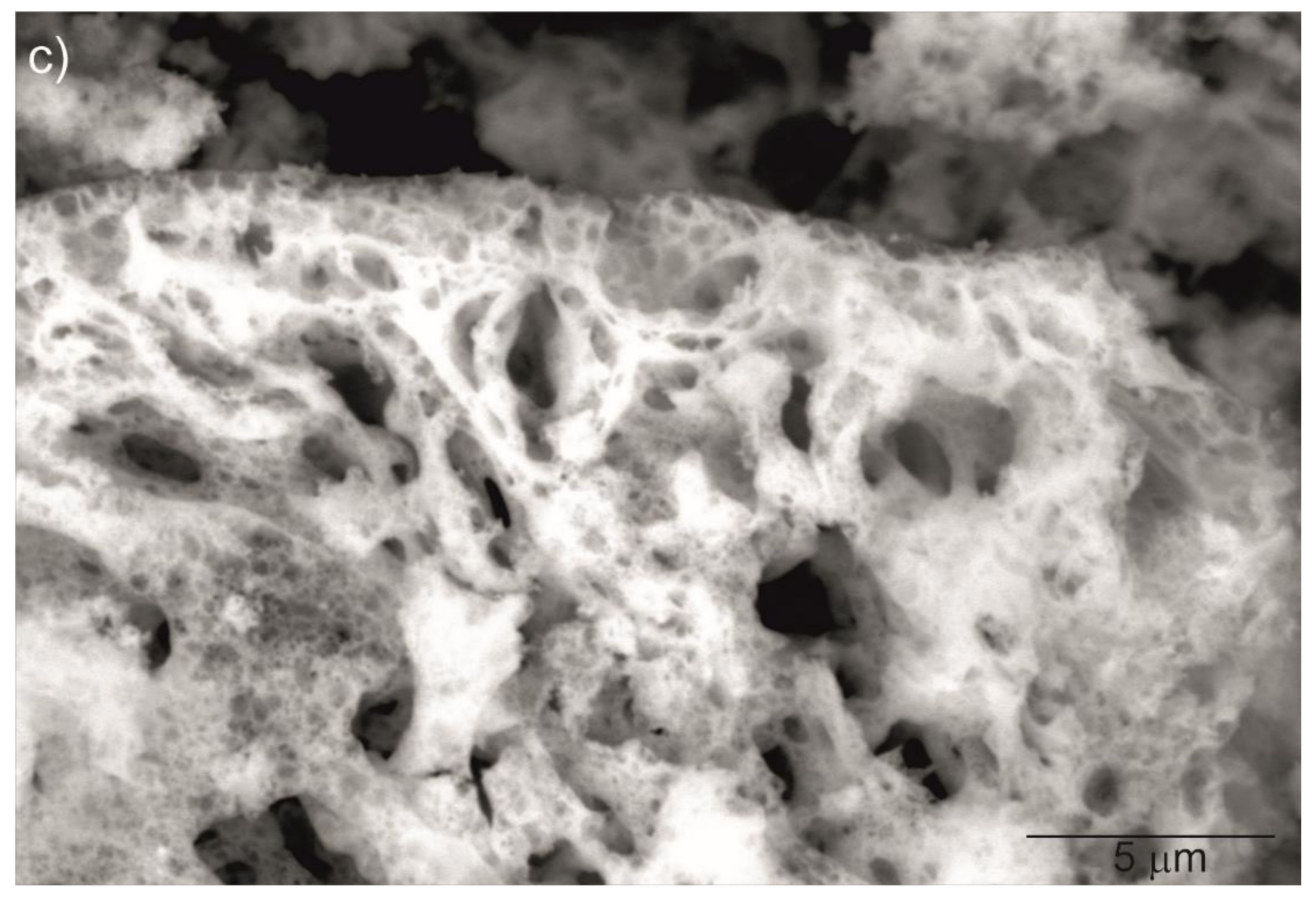
SEM micrograph of Y_2_O_3_ (**a**), La_0.1_Y_1.9_O_3_ (**b**) and La_0.1_Nd_0.12_Y_1.78_O_3_ (**c**) nanoparticles calcined at 973 K as prepared.

Figure 2 shows SEM images of the Y_2_O_3_, La_0.1_Y_1.9_O_3_ and La_0.1_Nd_0.12_Y_1.78_O_3_ nanoparticles calcined at 973 K. It can be seen that the nanosized particles are nearly spherical in shape and exhibit a propensity for forming multigrain agglomerates. All nanoparticles have a narrow particle size distribution and regular shape with a unimodal distribution. The BET surface area and SEM morphology reflected the fine-grained nature of the presented powders.

### 3.3. Bulk Ceramics Characterization

#### 3.3.1. XRD Analysis

**Figure 3:**
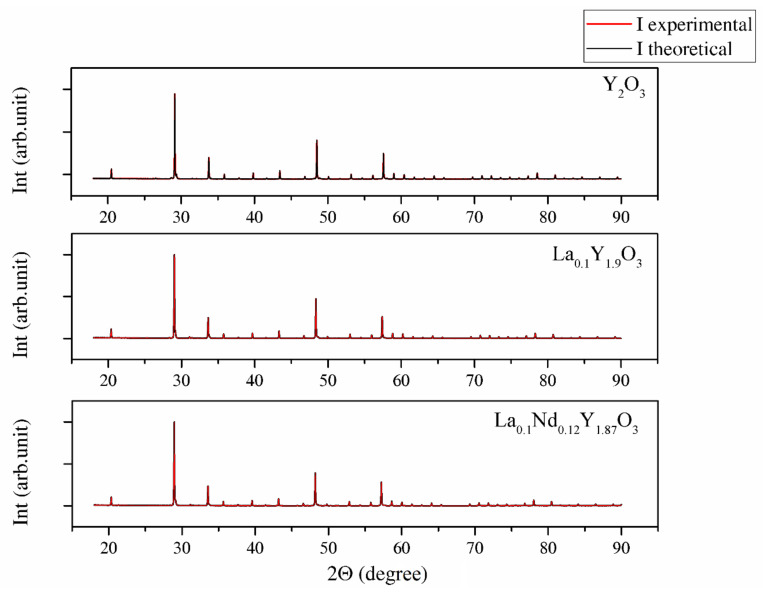
XRD patterns of Y_2_O_3_, La_0.1_Y_1.9_O_3_ and La_0.1_Nd_0.12_Y_1.78_O_3_ samples obtained via hot isostatic pressing for 2 h in argon at 1730 K.

The XRD patterns shown in Figure 3 were used to identify the phase of the investigated sample ceramics. The measured XRD patterns confirm only one phase with the standard PDF Card No. 86-1326 from the Inorganic Crystal Structure Database (ICSD). All reflections in the XRD patterns can be finely indexed to the cubic structure and Ia-3 group symmetry. No peaks from other phases were found, suggesting high phase purity and complete incorporation of doped ions into the yttria crystal lattice. The approximated values of the lattice parameter (*a*), unit cell volume *(V*), crystallite size *D_XRD_* and dislocation density (*ρ*) of the investigated bulk ceramics are summarized in Table 3.

The change in the lattice parameter of the sinters exhibited a similar trend compared with the powders (Table 3). Thus, the highest unit parameter was achieved for the La_0.1_Nd_0.12_Y_1.78_O_3_ sinter due to replacing the yttrium atoms (0.22 mol in total) with La, Nd ions in the yttria crystal lattice. The shift *2Θ* positions were shown (inset in Figure 3) by examining the reflection (222) closely. The Y_2_O_3_, La_0.1_Y_1.9_O_3_ and La_0.1_Nd_0.12_Y_1.78_O_3_ translucent ceramics achieved theoretical densities of 98.8%, 98.2% and 98.5%, respectively.

#### 3.3.2. SEM Observation

**Figure 4:**
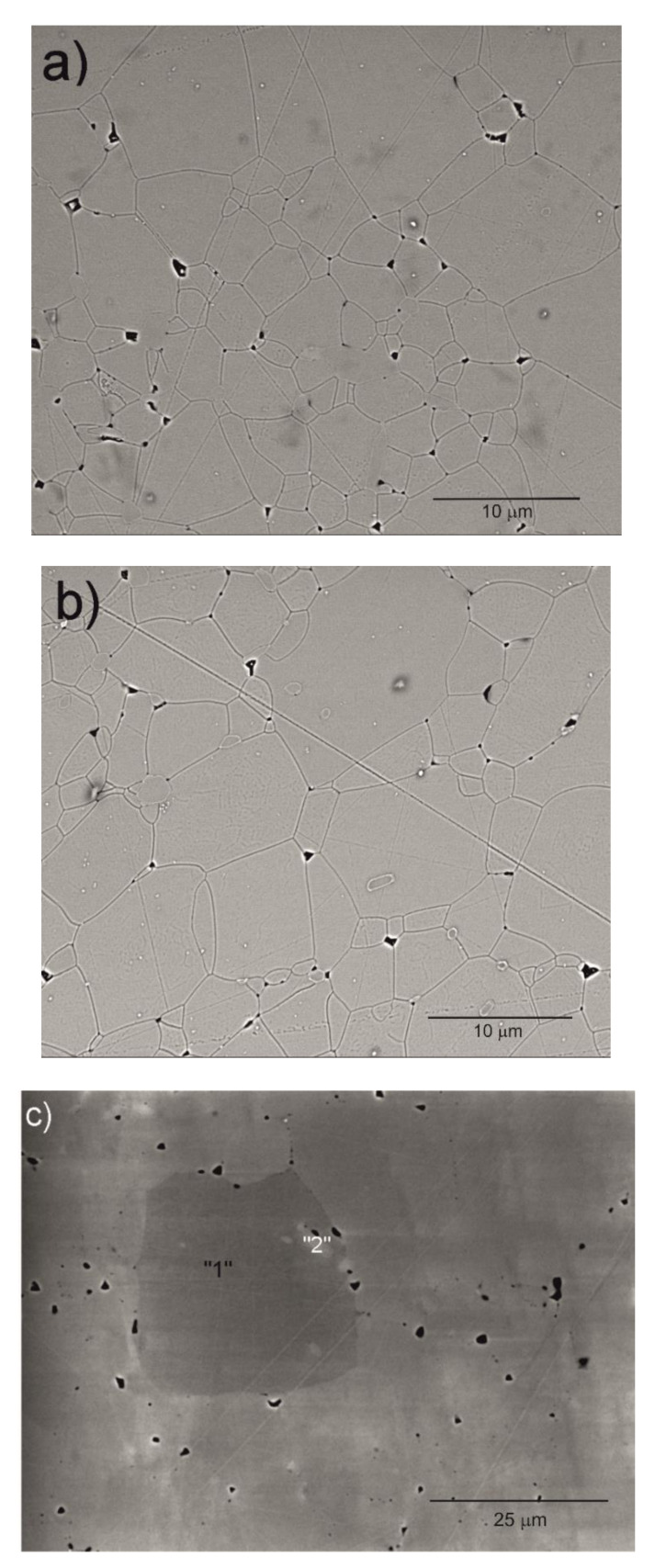
SEM micrograph of the surface of Y_2_O_3_ (**a**), La_0.1_Y_1.9_O_3_ (**b**) and La_0.1_Nd_0.12_Y_1.78_O_3_ (**c**) ceramic samples.

**Figure 5:**
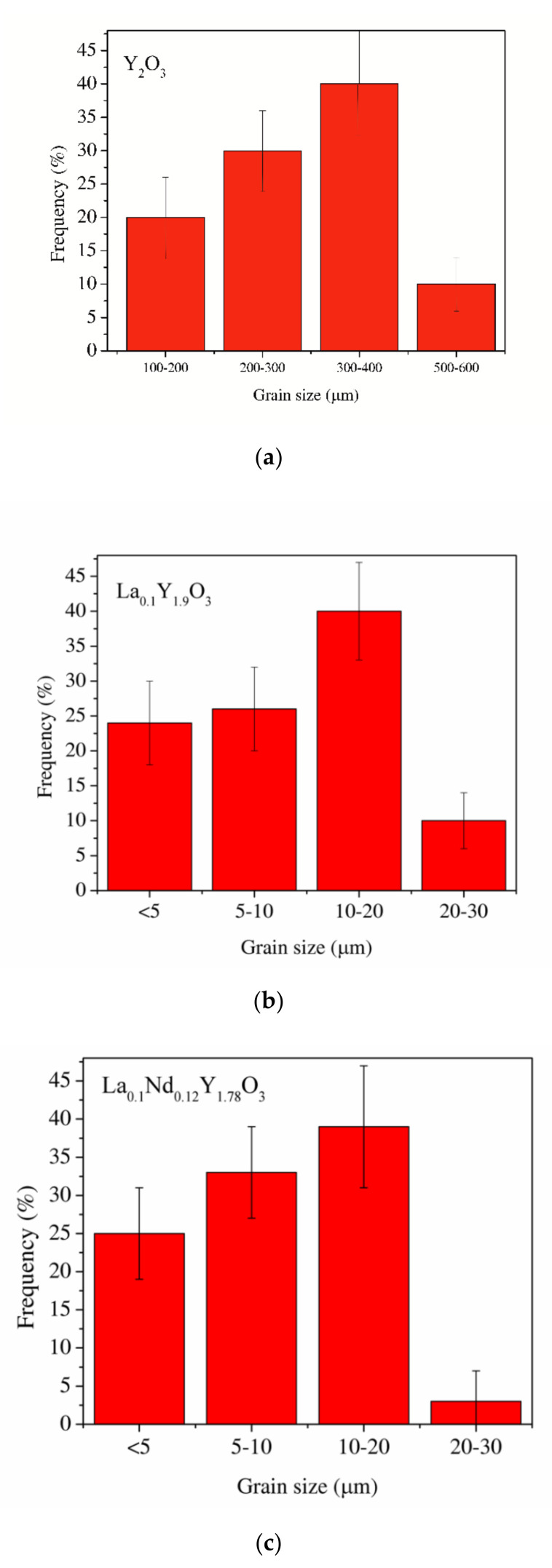
The average distribution of the diameters of grains of the Y_2_O_3_ (**a**), La_0.1_Y_1.9_O_3_ (**b**) and La_0.1_Nd_0.12_Y_1.78_O_3_ (**c**) ceramic samples.

SEM micrographs of the Y_2_O_3_, La_0.1_Y_1.9_O_3_ and La_0.1_Nd_0.12_Y_1.78_O_3_ ceramic samples are shown in Figure 4. A morphological observation of the surface of these sinters shows grains with a compact structure. It is noted that the microstructure is homogeneous and dependent on the chemical compounds. SEM scans of Y_2_O_3_ revealed that the grains are well developed. Almost all the pores between 1 and 2 µm can be observed at the grain boundaries; however, some pores can be found in the grains. The addition of lanthanum oxide to the yttria crystal lattice significantly increased the grain size from 5 to 10 µm. No secondary phase was present at the grain boundary of the La_0.1_Y_1.9_O_3_ crystals. Nearby, a similar microstructure is observed for La_0.1_Nd_0.12_Y_1.78_O_3_ (Figure 4c). As can be seen, the neodymium ions favor the formation of grains 10–20 µm in size. The average distribution of the diameters of grains with irregular structures was rather wide and unimodal for all the investigated samples (Figure 5). Aside from sparse isolated pores with a size below 1 µm, located in the grain interior, the majority of pores were found in the intergrain regions. As can be seen from Figure 4, the number of pores of the ceramic samples increased with increased doped ions in the matrix. Adding La or La/Nd ions changed the relative density of the HIP-treated material. These dopants presumably affect the crystallization of the final phase or alter the sintering kinetics. In the case of pure Y_2_O_3_, we observed the majority of pores with a maximum radius of ca. 5 µm. Several pores were found in the grain interior, and the majority of pores were located in the grain boundaries. 

**Figure 6:**
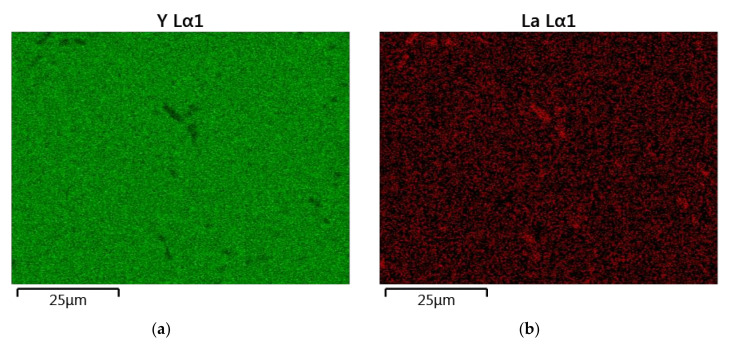

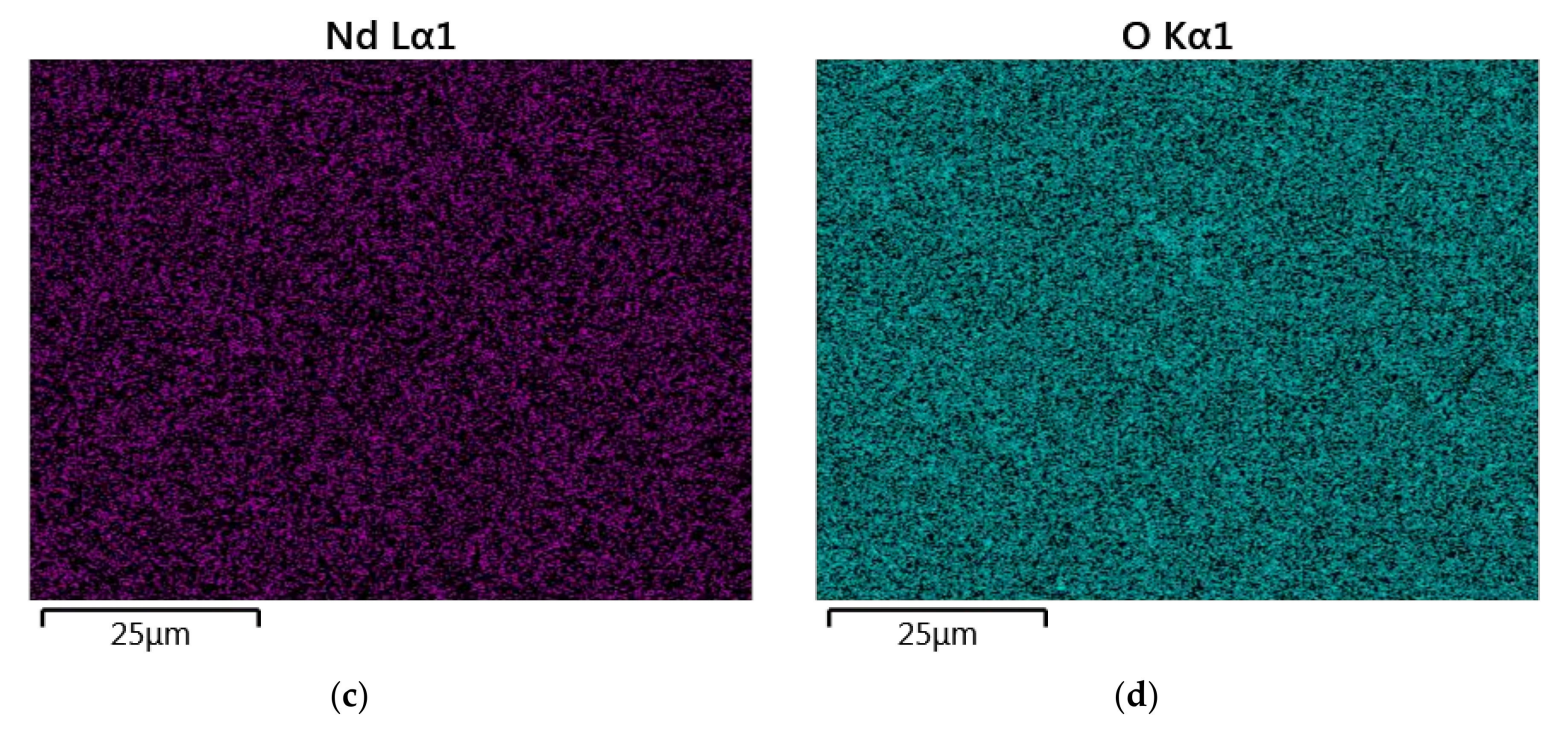
The map of the quantitative EDS chemical composition (at.%) analysis of Y (**a**), La (**b**), Nd (**c**) and O (**d**).

The results of the semiquantitative EDS chemical composition analysis (at.%) performed for the above-mentioned samples are presented in Table 4. The surface analysis of the chemical composition confirmed the high purity and expected qualitative composition of Y_2_O_3_. In the case of the La_0.1_Y_1.9_O_3_ sample, only pure Y_2_O_3_ at the grain exists. The map of the chemical composition of the La_0.1_Nd_0.12_Y_1.78_O_3_ sample revealed that some grains are rich in La_2_O_3_ compounds. Surface and major grains (area “1”) corresponded to the chemical composition of the La_0.1_Nd_0.12_Y_1.78_O_3_ sample (Figure 6, Table 2). The presence of the second type of grains rich in La_2_O_3_ was caused by the outdiffusion of La ions from the matrix grains during the HIP process. Based on the mapping by the EDS method, it can be concluded that some La-rich secondary phases present in small amounts in the HIP-treated sample have a low effect on the transparency of the material. This applies to the small inclusions of La-rich secondary phases, which are much greater than the wavelengths of visible light. Hence, according to Rayleigh and Mie’s theory, these inclusions exhibit a negligible effect on the scattering.

Nevertheless, this phase is not observed in the XRD diagram (Figure 3). This semiquantitative analysis (EDS) reveals that the chemical composition of bulk ceramics is very close to their stoichiometric composition (Table 2). 

#### 3.3.3. Raman Spectroscopy Analysis

**Figure 7:**
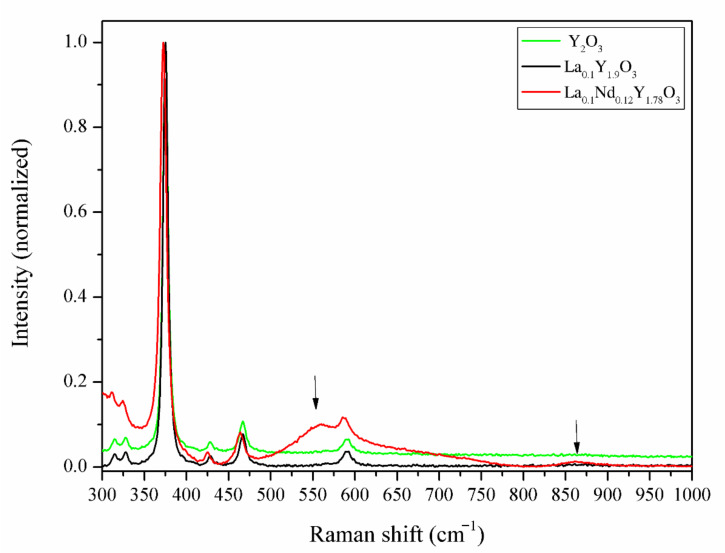
The Raman spectra of the Y_2_O_3_, La_0.1_Y_1.9_O_3_ and La_0.1_Nd_0.12_Y_1.78_O_3_ ceramic samples.

In order to verify the incorporation of La^3+^ and Nd^3+^ ions into the lattice structure of the host material, independent of the XRD experiment, Raman spectroscopy was carried out on the bulk samples. The recorded spectra are shown in Figure 7. The Raman experiments were conducted in the spectra range from 280 to 1000 cm^−1^, where the Raman-active modes of yttria exist. To compare the bulk ceramics, the individual Raman spectra were normalized. The Raman spectra of Y_2_O_3_ and La_0.1_Y_1.9_O_3_ showed a strong uniform phase in the crystal lattice of the host material without a secondary phase, impurities on the grain boundary or inside the grain. The Raman spectrum of the yttrium sinter exhibited a series of typical peaks with a major peak at 375 cm^−1^, which is related to the fundamental vibrational mode of cubic Y_2_O_3_. According to Replin [41], in the cubic yttria, there are 22 active modes in the Raman spectra. The irreducible group representations for the acoustical modes *Γ_ac_ = F_u_* and optical *Ã_op_* modes are:

*Ã_op_ = 14F_g_ + 4E_g_ + 4A_g_ + 5A_2u_ + 5E_u_ + 16F_u_* (5)

where *F_g_*, *E_g_* and *A_g_* are active Raman, *F_u_* is active *IR* and *A_2u_* and *E_u_* are inactive in theory. 

It is observed that only 14 modes were observed in the pure Y_2_O_3_ and La_0.1_Y_1.9_O_3_. This may be due to the likelihood of a band overlap, according to Ubaldini [42]. When both La and Nd ions were added into the pure yttria, additional Raman peaks arose at 1590, 1872, 2149 and 2352 cm^−1^, and in the range from 3400 to 4315 cm^−1^. Two peaks centered at 550 and 860 cm^−1^ are associated with the creation of the new phase rich in La^3+/^Nd^3+^ ions. It can be observed (Figure 7) that lanthanum and neodymium ions are not homogeneously distributed in the yttria matrix, which is consistent with the SEM and EDS observations. 

#### 3.3.4. UV–Vis–IR Absorbance Spectroscopy

**Figure 8:**
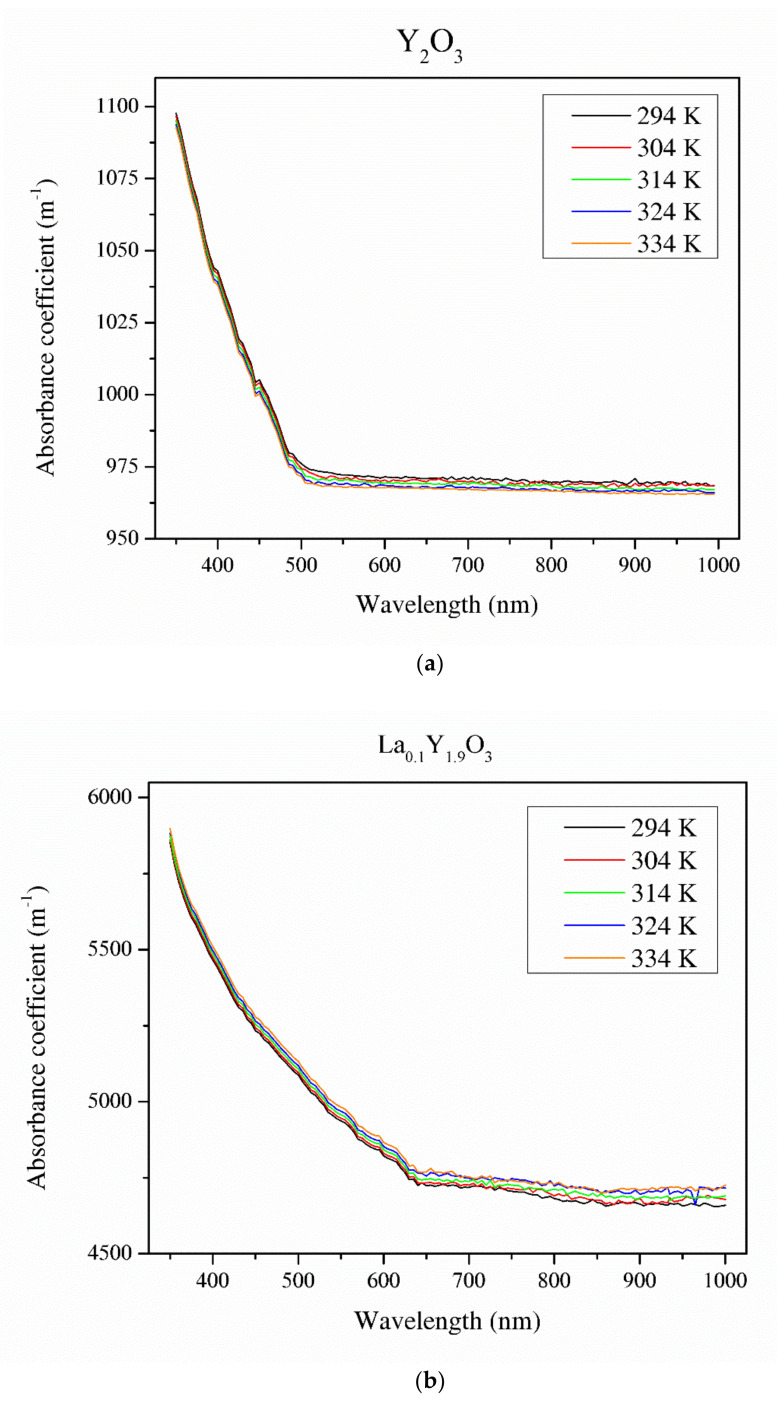

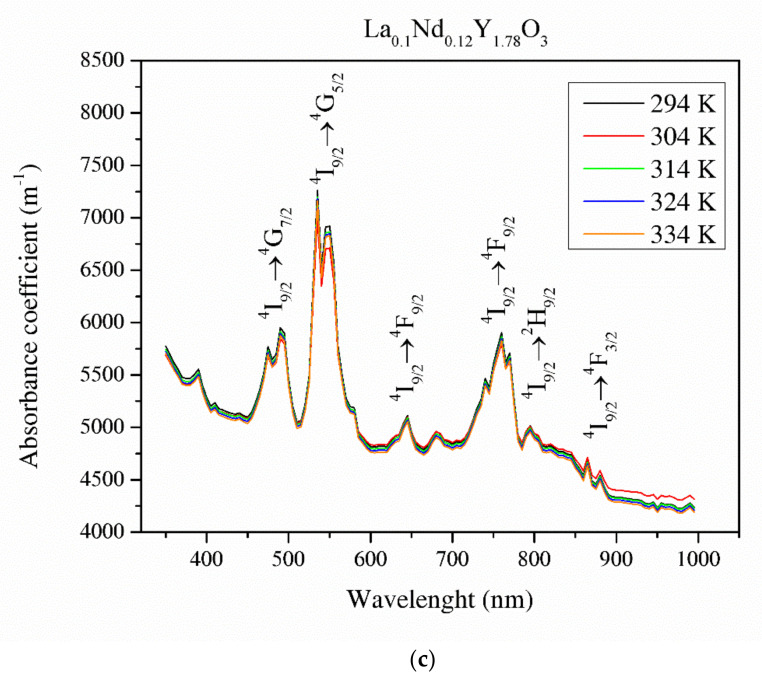
Thermal dependence of the absorbance coefficient of the Y_2_O_3_ (**a**), La_0.1_Y_1.9_O_3_ (**b**) and La_0.1_Nd_0.12_Y_1.78_O_3_ (**c**) translucent ceramics in the wavelength range of 350–1000 nm with a step of 1 nm.

Figure 8 illustrates the absorbance coefficient of Y_2_O_3_, La_0.1_Y_1.9_O_3_ and La_0.1_Nd_0.12_Y_1.78_O_3_ translucent ceramics at different temperatures. It is obvious that the transmittance of the samples decreases with increasing RE ion concentrations. Pure yttria achieved 56% transparency in the VIS region due to the presence of pores. La_0.1_Y_1.9_O_3_ has maximum transparency of 41% at 800 nm. Transmittance for the La_0.1_Nd_0.12_Y_1.78_O_3_ ceramics is above 40% in the visible-light range. These values are relatively low and can improve the value of the Verdet constant. These bulk ceramics indicate a higher level of transparency in the NIR wavelength range. This is affected by the nanometer range pores that activate relatively high scattering when light passes through the sintered ceramics. When the scatter center is smaller than the transmittance wavelength, the scatter intensity increases according to Rayleigh and Mie’s theories [43]. The absorption bands observed in the case of the La_0.1_Nd_0.12_Y_1.78_O_3_ sample between 500 and 550 nm in the transmittance spectrum comes from the transition of the Nd^3+^ ground state ^4^I_9/2_ to the Nd^3+^ excited state ^4^G_7/2_. The strong bands between 580 and 620 nm correspond to the transition of the Nd^3+^ ground state ^4^I_9/2_ to the Nd^3+^ excited state ^4^G_5/2_. The rest of the detected absorption bands in the VIS spectrum are correlated with the transition ground state ^4^I_9/2_ to the excited state ^4^F_9/2_ (680 nm), ^4^F_7/2_ (760 nm), ^2^H_9/2_ (820 nm) and ^4^F_3/2_ (900 nm) [44]. The absorption coefficient decreases with increasing temperature. Based on obtained experimental data and using the Tauc plot, the optical band gaps in the selected temperatures were calculated. 

#### 3.3.5. Optical Band Gap 

The optical band gaps (*E_g_*) of Y_2_O_3_, La_0.1_Y_1.9_O_3_ and La_0.1_Nd_0.12_Y_1.78_O_3_ were estimated using the UV–Vis–IR absorption coefficient spectra. The band gap energy *E_g_* is the energy difference between the highest occupied molecular orbital (top of the valence band) to the lowest unoccupied molecular orbital (bottom of the conduction band). The *E_g_* was computed using the Tauc equation:

*αhν = A × (hν−E_g_)^n^* (6)

where *A* is a parameter independent of the photon energy *E*, *Eg* is the optical band gap energy and the exponent *n* depends on the type of transition between bands, *h* is the Planck constant, α is the absorption coefficient and *ν* is the frequency of light. 

The energy band gaps of Y_2_O_3_, La_0.1_Y_1.9_O_3_ and La_0.1_Nd_0.12_Y_1.78_O_3_ were found to be 2.25, 2.52 and 2.73 eV, respectively. Due to the presence of absorption lines, the optical band gap values should be considered as estimated. This energy corresponds to the blue-light wavelength range of 400–480 nm. The reduction in the band gap with increasing temperature was due to the expansion in the strain factor [43,45]. The values optical band gaps increased by ca. 0.01 eV with increasing temperature (from 298 to 343 K) in all the investigated samples. 

#### 3.3.6. Luminescence Spectra

**Figure 9:**
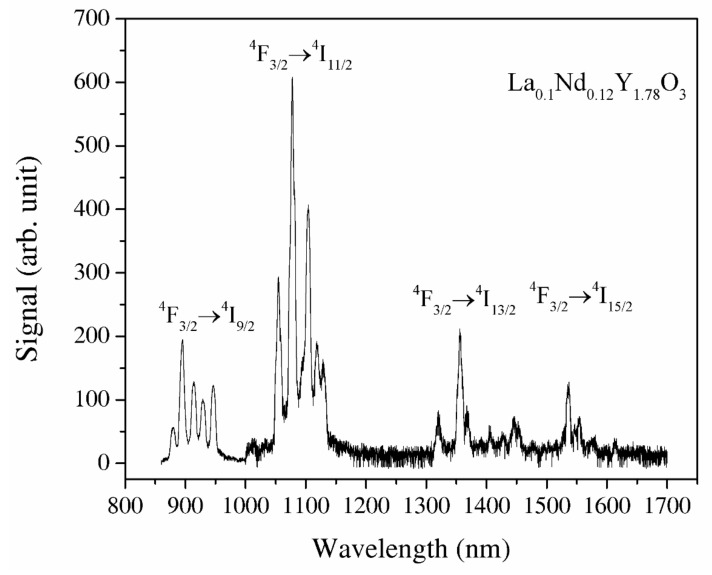
The luminescence spectra of the La_0.1_Nd_0.12_Y_1.78_O_3_ excited under 808 nm and the wavelength range of 850–1700 nm.

Y_2_O_3_ and La_0.1_Y_1.9_O_3_ do not indicate luminescence spectra under the excitation of an 808 nm laser. The recorded IR emission spectrum of the La_0.1_Nd_0.12_Y_1.78_O_3_ sinter in the spectral range of 900–1600 nm under the excitation of the NIR laser is displayed in Figure 9. The La, Nd-doped yttria indicates a bixbyite type. A primitive cell has 24 Y^3+^ noncentrosymetric sites (d sites with C_2_ symmetry) and 8 Y^3+^ centrosymmetric (b sites with S6 or C_3i_) sites. The RE group’s ions in Y_2_O_3_ occupy high- and low-symmetry sites. Energy transfer to Nd^3+^ ions via the yttria host lattice is a determinant of the luminescence behavior of rare-earth-doped materials. This energy transfer behavior is dependent on the structure and local microstructure of neodymium ions. The strong emission peaks between 1050 and 1150 nm are attributed to the ^4^F_3/2_ → ^4^I_11/2_ transitions and the second region with emission peaks between 1300 and 1400 nm ^4^F_3/2_ → ^4^I_13/2_ transitions, respectively. The emission bands with strong emission at 1525 nm are related to the transitions from the ^2^H_11/2_ and ^4^S_3/2_ excited states to the ^4^I_15/2_ ground state. The emission bands between 1500 and 1600 nm are attributed to the ^4^F_3/2_ → ^4^I_13/2_ transitions. According to Lei’s theory, the La^3+^ ions added into the host material doped with RE ions increased the luminescence properties of active ions [40]. Lanthanum ions increased the lattice parameter of the host material, so the local symmetry of the atoms around the active ions reduces. The f–f transitions of electric–dipole are parity-forbidden and become partially allowed when the rare-earth ion is situated at a low-symmetry site due to the intermixing of the intra-4f states with a higher electronic configuration [7]. As a result, the probability of f–f transitions increases, leading to an increase in the oscillator strength *f* [8,10]. 

#### 3.3.7. Luminescence Lifetime

**Figure 10:**
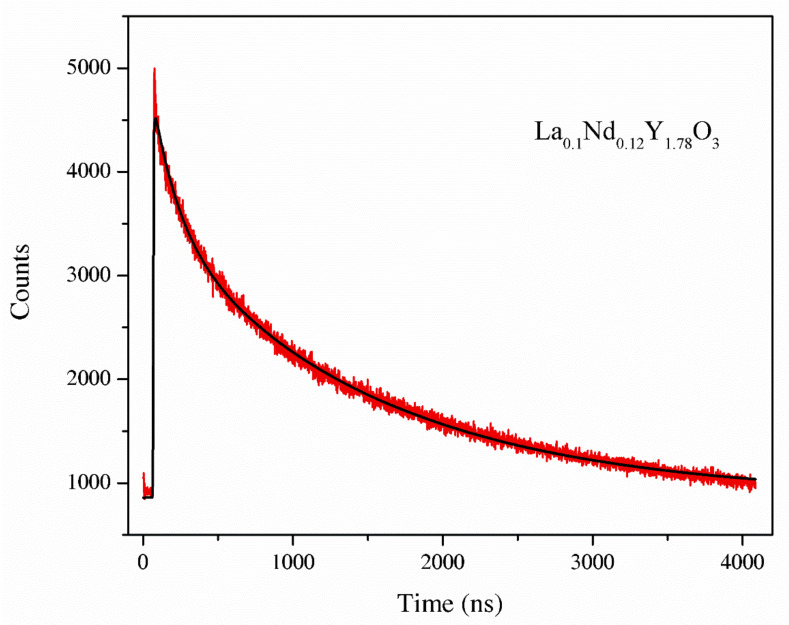
The luminescence decay curve of the La_0.1_Nd_0.12_Y_1.78_O_3_ sample.

The luminescence decay curve for the La_0.1_Nd_0.12_Y_1.78_O_3_ sinter is shown in Figure 10. The luminescence decay profile was adjusted by means of a double-exponential function. The decay curves were fitted according to Equation (7):

(7) $$y=y_{0}+A_{1}exp\left(-\frac{t}{T_{1}}\right)+A_{2}exp\left(-\frac{t}{T_{2}}\right)$$

where *t* is time, *A*, *y_0_* is the fit parameter and *T_1_*, *T_2_* are relaxation decay. 

Parameters *T_1_, T_2_* are the two components of the luminescence decay times that are attributed to the long and short component of the lifetime. The luminescence decay kinetics of the La_0.1_Nd_0.12_Y_1.78_O_3_ sinter exhibits two-component decay with decay times. Two relaxation decays corresponded Nd^3+^ ions in two different co-ordinations: the A-site and B-site of the cubic Y_2_O_3_ of 3.1 and 35 ns. The second component estimated was shorter than the value presented in [35], i.e., 300 ms. The measurement error is 0.1 ns.

#### 3.3.8. Verdet Constant Analysis

**Figure 11:**
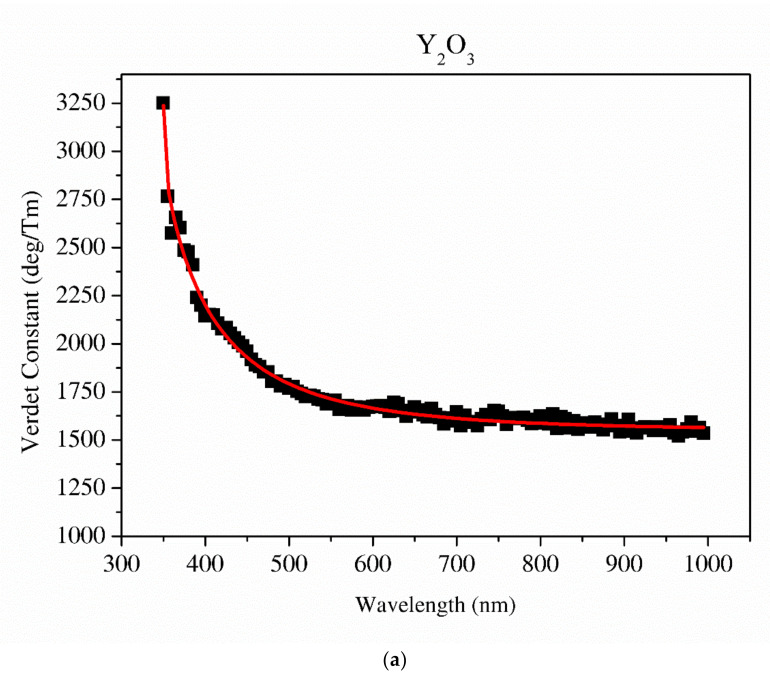

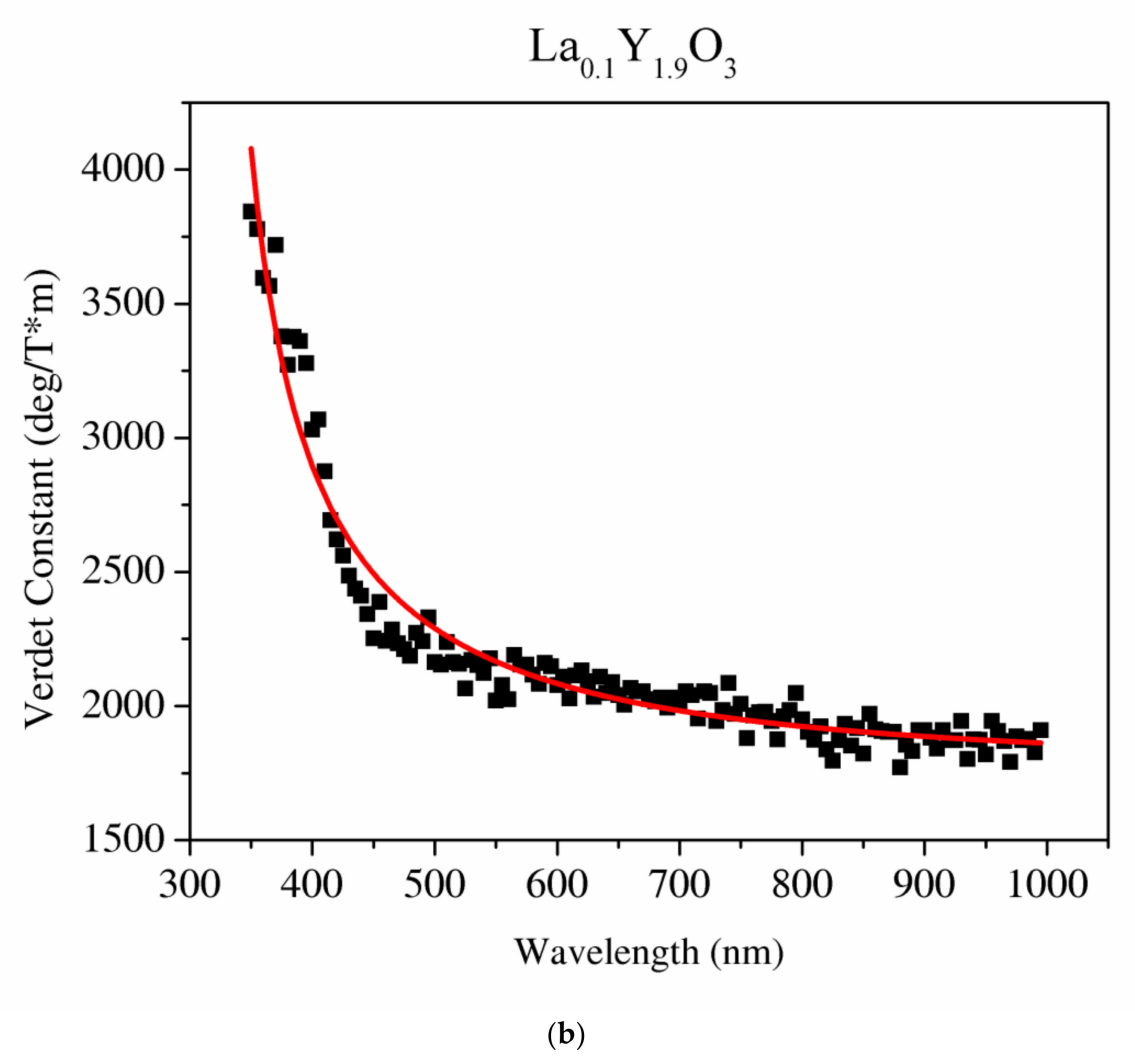
The Sellmaier curve fitted to the Verdet constant of Y_2_O_3_ (**a**) and La_0.1_Y_1.9_O_3_ (**b**).

**Figure 12:**
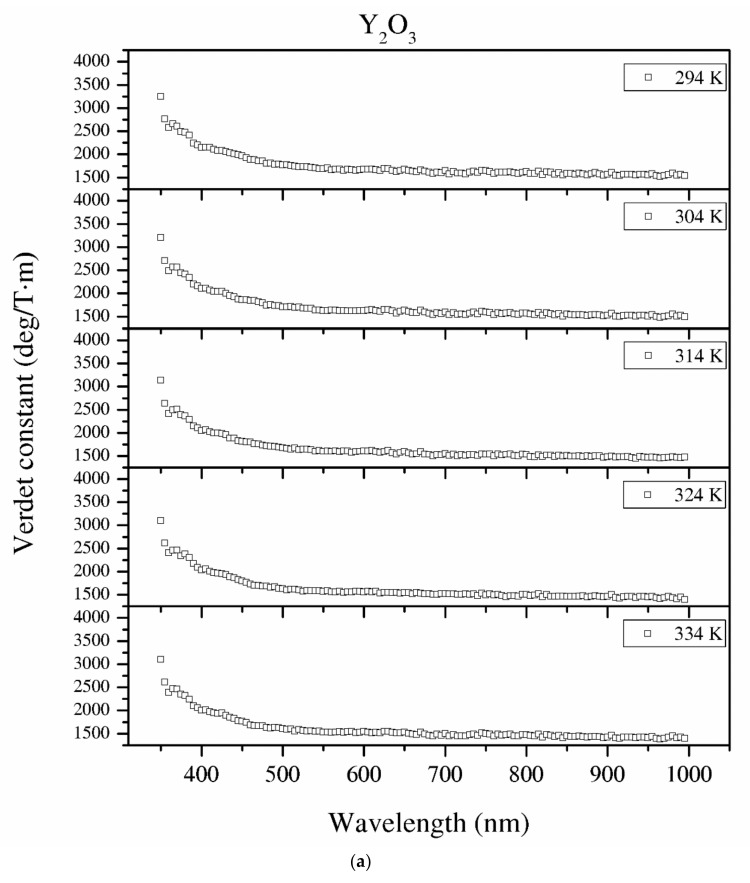

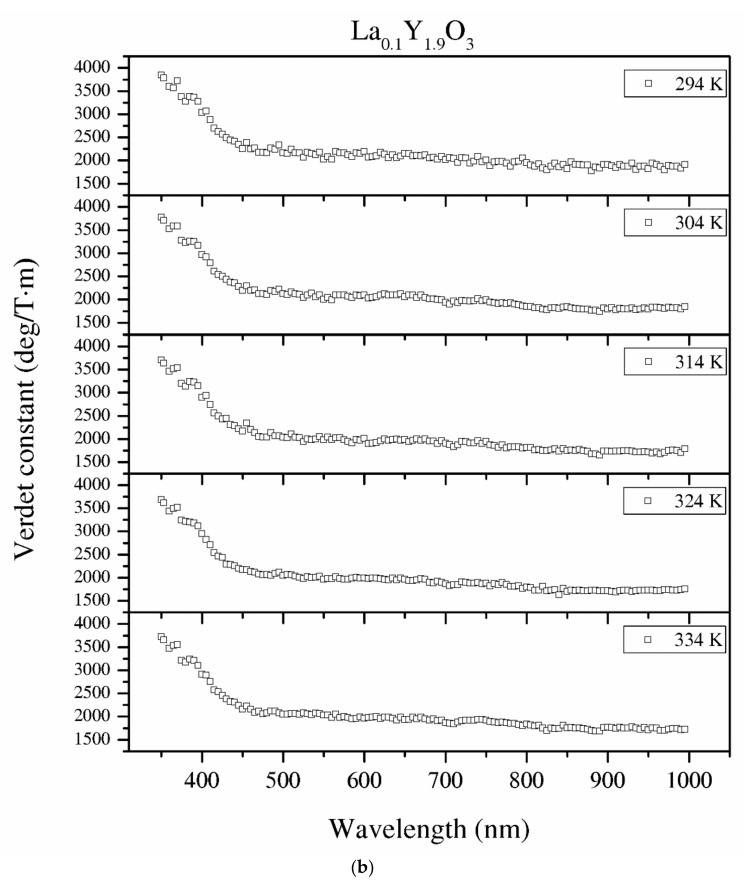

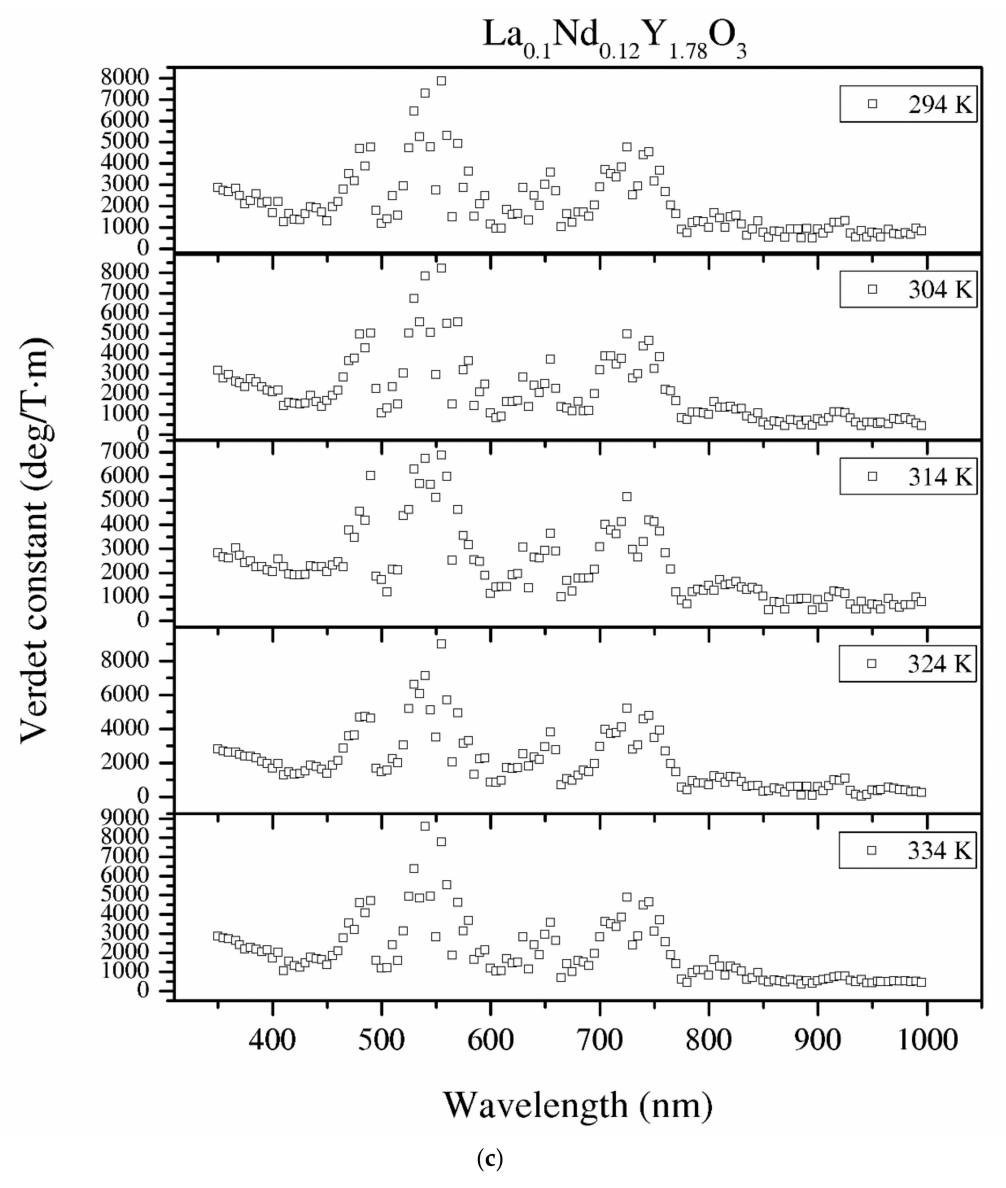
The Verdet constant of the Y_2_O_3_ (**a**), La_0.1_Y_1.9_O_3_ (**b**) and La_0.1_Nd_0.12_Y_1.78_O_3_ (**c**) samples as a function of the wavelength and temperature.

Results of the Verdet constant for the Y_2_O_3_, La_0.1_Y_1.9_O_3_ and La_0.1_Nd_0.12_Y_1.78_O_3_ transparent ceramics as a function of the wavelength and temperature are shown in Figure 11 and Figure 12. The Verdet constant *V* was calculated according to Equation (8):

*V = θ/BL* (8)

where *V* is the Verdet constant, *B* is the magnetic field, *L* is the length of the medium traversed and *θ* is the polarization rotary angle.

The relationship between the Verdet constant *V* and the dispersion of the refractive index *n* (precisely wavelength dispersion) is expressed as:

(9) $$V=-\frac{e\lambda }{2mc^{2}}\frac{dn}{d\lambda }$$

where *e* and *m* are the charge and mass of the free electron, *c* is the light speed and *dn/dλ* is the dispersion.

The detailed relationship between the wavelength dispersion and Verdet constant is described by Kramers–Kronig relations, which predict an interesting spectral variation of the Verdet constant across the absorption band (Equations (10) and (11)). The complex refractive index *n*, with real part *n*’ and imaginary part *n*’’ (called the extinction coefficient), is related to the complex relative permittivity *ε_r_*. From Figure 11 and Figure 12, it is obvious that the investigated samples indicate a positive value of the Verdet constant in the total spectral range. The variation of the Verdet constant with the wavelength for the Y_2_O_3_ sinter is shown in Figure 11. An evolution of the Verdet constant of La_0.1_Y_1.9_O_3_ showed similar characteristics to the pure Y_2_O_3_; however, the values are higher due to the presence of lanthanum ions (paramagnetic ions). The second reason for the increase in the Verdet constant is a higher dispersion, as predicted by the theory (Equation (9)). In contrast to the above-mentioned samples, La_0.1_Nd_0.12_Y_1.78_O_3_ shows many sharp peaks due to the absorption peaks associated with the optical transition in the Nd^3+^ ions, as predicted by the Kramers–Kronig relations (Equations (10) and (11)):

(10) $$\varepsilon_{r}^{\prime }\left(\omega \right)=1+\frac{2}{\pi }P\overset{\infty }{\underset{0}{\int }}\frac{\omega^{\prime }\varepsilon_{r}^{\prime \prime }\left(\omega^{\prime }\right)}{{\omega^{\prime }}^{2}-\omega^{2}}d\omega^{\prime }$$

(11) $$\varepsilon_{r}^{\prime \prime }\left(\omega \right)=-\frac{2\omega }{\pi }P\overset{\infty }{\underset{0}{\int }}\frac{\varepsilon_{r}^{\prime }\left(\omega^{\prime }\right)}{{\omega^{\prime }}^{2}-\omega^{2}}d\omega^{\prime }$$

where *ε′* and ε″ are the real and imaginary parts of the complex relative permittivity *ε_r_*, respectively. *ω′* is the integration variable, *P* represents the Cauchy principal value of the integral and the singularity at *ω′* = *ω* is avoided.

The results can be explained via the quantum theory of Faraday rotation, taking into account the influence of effective magnetic fields on the splitting of the ground and excited multiplets of rare-earth ions and the mixing of wave functions of the ground and excited multiplets of rare-earth ions.

It can be seen that values of the Verdet constant in Y_2_O_3_, La_0.1_Y_1.9_O_3_ and La_0.1_Nd_0.12_Y_1.78_O_3_ at 450 nm reach 2500 (deg/T·m), 3500 (deg/T·m), 3600 (deg/T·m), respectively. The Verdet constant of the investigated transparent ceramics is lower than that shown in commonly used garnet crystals (TSAG is 8949 rad/T·m at 633 nm, YIG is 380 rad/T·m at 780 nm and YAG is 11 172 rad/T·m at 633 nm) [46,47,48]. 

The experimental data of the Verdet constant vs. the wavelength were fitted to the Sellmeier-type equation given in Equation (12):

(12) $$n^{2}=1+\frac{B_{1}\lambda^{2}}{\lambda^{2}-C_{1}}+\frac{B_{2}\lambda^{2}}{\lambda^{2}-C_{2}}+\frac{B_{3}\lambda^{2}}{\lambda^{2}-C_{3}}$$

where *n* is the refractive index, *λ* is the wavelength and *B_1,_ B_2,_ B_3_* and *C_1_, C_2_, C_3_* are experimentally determined Sellmeier coefficients.

The Sellmaier curve was successfully fitted to Y_2_O_3_ and La_0.1_Y_1.9_O_3_ experimental data in the spectrum range from 350 to 1000 nm as shown in Figure 11. Due to the absorption bands in the La_0.1_Nd_0.12_Y_1.78_O_3_ sample, it was impossible to fit these parameters precisely. The parameters of the Sellmaier equation for Y_2_O_3_ and La_0.1_Y_1.9_O_3_ are shown in Table 5. In the case of the La_0.1_Y_1.9_O_3_ sample, only the first two parameters of the Sellmaier equation were computed. The matching factor for Y_2_O_3_ and La_0.1_Y_1.9_O_3_ is high and *R^2^* is equal to 0.989 and 0.969, respectively. 

In order to determine the influence of the temperature on the Verdet constant, tests were carried out at selected temperatures.

#### 3.3.9. Verdet Constant in the Function of Temperature

Temperature dependence of the Verdet constant obtained from the measured Faraday phenomena is shown in Figure 12 for the Y_2_O_3_, La_0.1_Y_1.9_O_3_ and La_0.1_Nd_0.12_Y_1.78_O_3_ samples as a function of the wavelength. As can be seen from Figure 12. the Verdet constants decreased with increasing temperature. The temperature dependence of the Verdet constant in the case of all samples can be described as:

(13) $$V_{T}=A\times T+B$$

where *T* is the temperature of the materials and *A* and *B* are the material-specific constants.

For Y_2_O_3,_ La_0.1_Y_1.9_O_3_ and La_0.1_Nd_0.12_Y_1.78_O_3_, the parameters for 450 nm were estimated as *A* = −4.5 and *B* = 3268, and *A* = −2.1, *B* = 2824 and *A* = −2.0 and *B* = 2176, respectively. The Verdet constant decreased above 2 deg/T·m for every 1 K. The total Verdet constant is the result of the algebraic sum of the components derived from the two types of ions: paramagnetic and diamagnetic. Paramagnetic ions depend on the wavelength *λ* and temperature *T*, and the diamagnetic ions depend on *λ* and are very weak on *T*. The presented parameters are indicative of the narrow temperature range and relatively high measured errors. 

Both a tunable optical parametric oscillator laser and a high-efficiency cryostat are required to conduct highly accurate measurements. Nevertheless, these studies should be supported by further theoretical and experimental studies.

#### 3.3.10. Figure of Merit of the Verdet Constant

From the Verdet constant (Figure 11) and absorbance coefficient *α* (Figure 8) obtained at room temperature, the magneto-optical figure of merit (FOM) was calculated by the following relation:

*FOM = V/α* (14)

**Figure 13:**
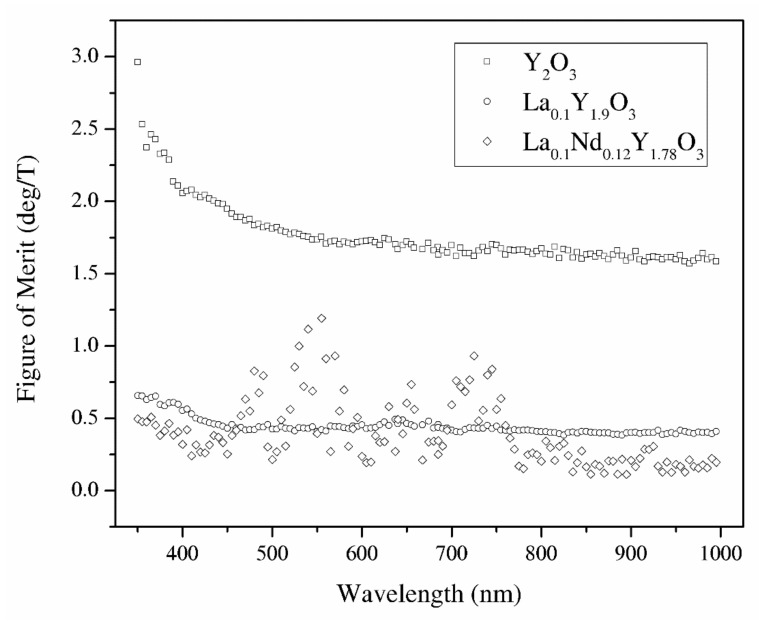
The magneto-optical figure of merit of the Y_2_O_3_, La_0.1_Y_1.9_O_3_ and La_0.1_Nd_0.12_Y_1.78_O_3_ translucent ceramics in the wavelength range of 350–1000 nm.

The magneto-optical figure of merit (FOM) of the above-mentioned samples is shown in Figure 13. The highest values of the magneto-optical FOM in the total spectrum achieve pure Y_2_O_3_ because of the low absorbance coefficient. However, a higher loss and lower FOM are observed in the RE-doped yttria as well as the increasing Verdet constant. Sample La_0.1_Nd_0.12_Y_1.78_O_3_ shows the highest values of the MO effect in the wavelength region where they show absorption bands. In particular, the sample exhibits a Verdet constant larger than 25 deg/m at around 400 nm with a magnetic field of 0.01 T. Considering the experimental errors in the Faraday rotation angle and absorbance coefficient, the magneto-optical figure of merit measurement error is 10%. 

## 4. Conclusions

Y_2_O_3_, La_0.1_Y_1.9_O_3_ and La_0.1_Nd_0.12_Y_1.78_O_3_ nanophosphors were obtained by a wet chemistry method. These powders were sintered by the hot isostatic pressed method. It was found that lanthanum and neodymium compensation promotes grain growth resulting in better crystallization. The samples have a relatively high absorption coefficient level in the VIS–IR region; however, the change of sintering condition obtains high-transparency samples. To improve the optical performance of pure and doped Y_2_O_3_ bulk ceramics, it is necessary to reduce the pores. SEM observation revealed the presence of grains, which are consistent with the La_2_O_3_ phase. The bulk ceramics indicate relatively high values of the Verdet constant in the whole analyzed spectrum as well as low thermal dependence. The magneto-optical figure of merit for Y_2_O_3_, La_0.1_Y_1.9_O_3_ and La_0.1_Nd_0.12_Y_1.78_O_3_ at 535 nm and room temperature is 1.75, 0.43, and 0.72 (deg/T), respectively. Thus, Y_2_O_3_ can be a promising candidate as a host material for magneto-optical devices in the VIS region after doped paramagnetic ions (Tb, Dy). The La_0.1_Nd_0.12_Y_1.78_O_3_ sinter has too many absorption lines in the VIS region and could be used as a magneto-optical material in the IR telecommunication region where absorbance bands are not present. The Verdet constant across the absorption band is interesting. 

## Figures and Tables

**Table 1:** List of the yttrium-oxide-based materials studied.

Sinter	Shape	Dimensions
Y_2_O_3_	Cylinder:	5 mm in Diameter, 0.8 mm Thickness
La_0.1_Y_1.9_O_3_	Cylinder:	5 mm in Diameter, 0.68 mm Thickness
La_0.1_Nd_0.12_Y_1.78_O_3_	Cylinder:	5 mm in Diameter, 0.59 mm Thickness

**Table 2:** Lattice constants *d_hkl_*, crystallite size *a*, unit cell volume *V*, crystallite size *D*_XRD_, dislocation densities *ρ* and BET parameters of the Y_2_O_3_, La_0.1_Y_1.9_O_3_ and La_0.1_Nd_0.12_Y_1.78_O_3_ powders after 10 h of calcination in air at 973 K.

Powder	*d_hkl_* (222) (Å)	*a* (Å)	*V* (Å^3^)	*D_xrd_* (nm)	*ρ* (10^14^ m^−2^)	*BET* (m^2/^g)
Y_2_O_3_	3.05	10.5718	1181.5170	15	11.1	3.21
La_0.1_Y_1.9_O_3_	3.06	10.6238	1199.0565	11	44.4	3.24
La_0.1_Nd_0.12_Y_1.78_O_3_	3.07	10.6659	1213.3679	10	100	3.36

**Table 3:** Lattice constants *a*, unit cell volume *V*, crystallite size *D_XRD_* and dislocation densities *ρ* of the Y_2_O_3_, La_0.1_Y_1.9_O_3_ and La_0.1_Nd_0.12_Y_1.78_O_3_ bulk samples after the hot isostatic pressing (HIP) process.

	*a* (Ǻ)	*V* (Ǻ^3^)	*D_XRD_* (nm)	*ρ* 10^14^ (m^−2^)
Y_2_O_3_	10.61	1194	95.06	11.01
La_0.1_Y_1.9_O_3_	10.63	1211	87.45	1.32
La_0.1_Nd_0.12_Y_1.78_O_3_	10.66	1211	69.30	2.10

**Table 4:** Chemical composition of the studied powders as determined by EDS analysis. The carbon contamination was not shown.

	Y (Wt.%)	La (Wt.%)	Nd (Wt.%)	O (Wt.%)
Y_2_O_3_	76.1	–	–	19.1
La_0.1_Y_1.9_O_3_	72.1	4.4	–	18.4
La_0.1_Nd_0.12_Y_1.78_O_3_	70.6	4.9	6.2	18.3
La_0.1_Nd_0.12_Y_1.78_O_3_ point “1”	71.5	4.3	6.1	18.1
La_0.1_Nd_0.12_Y_1.78_O_3_ point “2”	37.6	30.2	8.6	23.7

**Table 5:** The Sellmaier parameters of the Verdet constant of Y_2_O_3_, La_0.1_Y_1.9_O_3_ samples.

	*B_1_*	*B_2_*	*B_3_*	*C_1_*	*C_2_*	*C_3_*
Y_2_O_3_	31.58	1.28	23.39	275.45	349.2	366.51
La_0.1_Y_1.9_O_3_	35.30	23.93	0	285.44	291.88	0

## References

[1] Zhu H., Zhang Y., Yin D., Wang J., Duan Y., Zhang J., Liu P., Tang D. (2018). Highly efficient CW operation of a diode pumped Nd:Y2O3 ceramic laser. Opt. Mater. Express.

[2] Secu M., Secu C., Bartha C. (2016). Crystallization and luminescence properties of a new Eu 3+ -doped LaOCl nano-glass-ceramic. J. Eur. Ceram. Soc..

[3] Yanagida T. (2018). Inorganic scintillating materials and scintillation detectors. Proc. Jpn. Acad. Ser. B.

[4] Greskovich C., Duclos S. (1997). Ceramic Scintillators. Annu. Rev. Mater. Res..

[5] Chapman M.G. (2005). Thermal post-fabrication processing of Y2O3:tm ceramic scintillators. Master’s Thesis.

[6] Irankhah R., Rahimipour M.R., Zakeri M., Razavi M., Zakeri M. (2018). Optical and mechanical properties of transparent YAG ceramic produced by reactive spark plasma sintering (RSPS). Mater. Res. Express.

[7] Patra A., Sominska E., Ramesh S., Koltypin Y., Zhong Z., Minti H., Reisfeld R., Gedanken A. (1999). Sonochemical Preparation and Characterization of Eu2O3and Tb2O3Doped in and Coated on Silica and Alumina Nanoparticles. J. Phys. Chem. B.

[8] Cherepy N. (2010). Transparent ceramic scintillators for gamma-ray spectroscopy and radiography. SPIE Newsroom.

[9] Dutta D.P., Tyagi A. (2009). Inorganic Phosphor Materials for Solid State White Light Generation. Solid State Phenom..

[10] Hubbard K.J., Schlom D.G. (1996). Thermodynamic stability of binary oxides in contact with silicon. J. Mater. Res..

[11] Aghazadeh M., Ghaemi M., Golikand A.N., Yousefi T., Jangju E. (2011). Yttrium Oxide Nanoparticles Prepared by Heat Treatment of Cathodically Grown Yttrium Hydroxide. Isrn Ceram..

[12] Yu M., Chen X., Mei G. (2018). Hydrothermal synthesis and luminescent properties of Y2O3:Eu3+ from waste phosphors. Results Phys..

[13] Srinivasan R., Yogamalar N.R., Elanchezhiyan J., Joseyphus R.J., Bose A.C. (2010). Structural and optical properties of europium doped yttrium oxide nanoparticles for phosphor applications. J. Alloy Compd..

[14] Chen J., Huang J., Huang C., Sun X. (2017). Preparation of nanoscaled yttrium oxide by citrate precipitation method. J. Rare Earth.

[15] Kang Y.C., Bin Park S., Lenggoro I.W., Okuyama K. (1999). Preparation of nonaggregated Y2O3: Eu phosphor particles by spray pyrolysis method. J. Mater. Res..

[16] Djuricic B., Kolar D., Memic M. (1992). Synthesis and properties of Y2O3 powder obtained by different methods. J. Eur. Ceram. Soc..

[17] Gajović A., Tomasic N., Djerdj I., Su D., Furic K. (2008). Influence of mechanochemical processing to luminescence properties in Y2O3 powder. J. Alloy Compd..

[18] Gan L., Park Y.-J., Zhu L.-L., Go S.-I., Kim H., Kim J.-M., Ko J.-W. (2017). Fabrication and properties of La2O3-doped transparent yttria ceramics by hot-pressing sintering. J. Alloy Compd..

[19] Ikesue A., Kamata K., Yoshida K. (2010). ChemInform Abstract: Synthesis of Transparent Nd-Doped HfO2-Y2O3 Ceramics Using HIP (hot isostatic pressing). Chemins.

[20] Majima K., Niimi N., Watanabe M., Katsuyama S., Nagai H. (1993). Effect of LiF addition on the preparation of transparent Y2O3 by the vacuum hot pressing method. J. Alloy Compd..

[21] Ahsanzadeh-Vadeqani M., Razavi R.S. (2016). Spark plasma sintering of zirconia-doped yttria ceramic and evaluation of the microstructure and optical properties. Ceram. Int..

[22] Kruk A., Jany B.R., Owczarczyk K., Madej D. (2019). On the possibility of using arc plasma melting technique in preparation of transparent yttria ceramics. Opt. App..

[23] Qiu J., Tanaka K., Hirao K. (2005). Preparation and Faraday Effect of Fluoroaluminate Glasses Containing Divalent Europium Ions. J. Am. Ceram. Soc..

[24] Snetkov I., Permin D.A., Balabanov S.S., Palashov O.V. (2016). Wavelength dependence of Verdet constant of Tb3+:Y2O3 ceramics. Appl. Phys. Lett..

[25] Serber R. (1932). The Theory of the Faraday Effect in Molecules. Phys. Rev..

[26] Hwang Y., Kim H., Cho S., Kim T., Um Y., Park H., Jeen G. (2006). Magnetic and magneto-optical properties in diluted magnetic semiconductors: Cd1−x−yMnxFeyTe single crystals. J. Magn. Magn. Mater..

[27] Castera J., Hepner G. (1977). Isolator in integrated optics using the Faraday and Cotton-mouton effects. IEEE Trans. Magn..

[28] Deeter M.N., Rose A.H., Day G.W. (1991). Faraday-Effect Magnetic Field Sensors Based on Substituted Iron Garnets.

[29] Yin H., Zhao G., Liu P., Wang S., Guo H. (2014). Preparation and performance of magneto-optical glasses doped with Tb3+/Dy3+. J. Wuhan Univ. Technol. Sci. Ed..

[30] Kruk A. (2017). Optical and structural properties of arc melted Ce or Pr –doped Y2O3 transparent ceramics. Ceram. Int..

[31] Zhang W., Guo F., Chen J. (2007). Growth and characterization of Tb3Ga5−xAlxO12 single crystal. J. Cryst. Growth.

[32] Qiu J., Tanaka K., Sugimoto N., Hirao K. (1997). Faraday effect in Tb3+-containing borate, fluoride and fluorophosphate glasses. J. Non-Cryst. Solids.

[33] Mei M., Cao L.L., He Y., Zhang R.R., Guo F.Y., Zhuang N.F., Chen J.Z. (2011). Growth and Magneto-Optical Properties of CaTbAlO4 Crystal. Adv. Mater. Res..

[34] Starobor A., Mironov E., Palashov O. (2019). High-power Faraday isolator on a uniaxial CeF3 crystal. Opt. Lett..

[35] Kruk A., Brylewski T., Mrózek M. (2019). Optical and magneto-optical properties of Nd0.1La0.1Y1.8O3 transparent ceramics. J. Lumin.

[36] Scherrer P. (1918). Nachrichten von der Gesellschaft der Wissenschaften zu Göttingen. Math.-Phys. Klasse.

[37] Kruk A., Mrózek M. (2020). The measurement of Faraday effect of translucent material in the entire visible spectrum. Measurements.

[38] Kakihana M. (1996). Invited review “sol-gel” preparation of high temperature superconducting oxides. J. Sol.-Gel Sci. Technol..

[39] Sillén L.G., Martell A.E. (1965). Stability Constants of Metallic-ion Complexes. Soil Sci..

[40] Lei R., Wang H., Xu S., Tian Y., Yang Q. (2016). Combustion synthesis and enhanced 1.5 μm emission in Y2O3:Er3+ powders codoped with La3+ ions. J. Rare Earths.

[41] Repelin Y., Proust C., Husson E., Beny J. (1995). Vibrational Spectroscopy of the C-Form of Yttrium Sesquioxide. J. Solid State Chem..

[42] Ubaldini A., Carnasciali M.M. (2008). Raman characterisation of powder of cubic RE2O3 (RE=Nd, Gd, Dy, Tm, and Lu), Sc2O3 and Y2O3. J. Alloy. Compd..

[43] Li X., Xie L., Zheng X. (2012). The comparison between the Mie theory and the Rayleigh approximation to calculate the EM scattering by partially charged sand. J. Quant. Spectrosc. Radiat. Transf..

[44] Walsh B.M., McMahon J.M., Edwards W.C., Equall R.W., Hutcheson R.L., Barnes N.P. (2002). Spectroscopic characterization of Nd:Y2O3: Application toward a differential absorption lidar system for remote sensing of ozone. J. Opt. Soc. Am. B.

[45] O’Donnell K.P., Chen X. (1991). Temperature dependence of semiconductor band gaps. Appl. Phys. Lett..

[46] Hou X., Zhou S., Jia T., Lin H., Teng H. (2011). Effect of Nd concentration on structural and optical properties of Nd:Y2O3 transparent ceramic. J. Lumin..

[47] Ivanov I.A., Karimov D., Snetkov I., Palashov O., Kochurikhin V., Masalov A., Fedorov V., Ksenofontov D., Kabalov Y. (2017). Study of the influence of Tb-Sc-Al garnet crystal composition on Verdet constant. Opt. Mater..

[48] Weller L., Kleinbach K.S., Zentile M.A., Knappe S., Hughes I.G., Adams C.S. (2012). Optical isolator using an atomic vapor in the hyperfine Paschen–Back regime. Opt. Lett..

